# Exploring a Synergistic Approach: Dual GLP-1 Agonist Combined with Degludec Basal Insulin for Early Type 1 Diabetes Treatment and its Impact on Albumin-Insulin Producing Cells Expression

**DOI:** 10.34172/apb.2024.040

**Published:** 2024-03-18

**Authors:** Amr Ahmed, Maher Monir. Akl

**Affiliations:** ^1^The public health department, MSc degree in gynecology and obstetrics, Riyadh First Health Cluster, Ministry of Health, Saudia Arabia.; ^2^Department of Chemistry, Faculty of Science, Mansoura University, 35516, Mansoura, Egypt.

**Keywords:** Early type 1 diabetes, Type 2 diabetes, Dual GLP-1 agonist, Degludec basal insulin, GLP-1 agonists, Cell proliferation, Insulin-producing cells, Anti-diabetic effects, Adult human hepatic tissue, Modified GLP-1 derivat

## Abstract

**Purpose:**

This manuscript explores the potential of dual glucagon-like peptide 1 (GLP-1) agonists combined with degludec basal insulin as a treatment approach for early type 1 diabetes. The study aims to evaluate the efficacy and mechanistic impact of semaglutide, a GLP-1 agonist, on newly diagnosed type 1 diabetes patients.

**Methods:**

A retrospective analysis was conducted to assess the effects of semaglutide on individuals with early type 1 diabetes. The analysis focused on the elimination of prandial and basal insulin, changes in C-peptide levels, and overall glycemic control. The study also examined the potential for GLP-1 agonists to protect residual beta cells, stimulate cell proliferation, and reprogram liver cells into insulin-producing cells. Additionally, the modification of GLP-1 agonists with albumin ligands to extend their half-life and enhance their anti-diabetic effects was investigated.

**Results:**

The findings demonstrate the elimination of both prandial and basal insulin requirements, an increase in C-peptide levels, and improved glycemic control among the patients. Despite the positive outcomes, the study’s retrospective nature and absence of a control group highlight the necessity for larger, prospective trials.

**Conclusion:**

GLP-1 agonists show considerable potential in the management of type 1 diabetes by protecting residual beta cells, promoting cell proliferation, and reprogramming hepatic cells. The integration of modified GLP-1 agonists with albumin ligands could further enhance these effects. The manuscript underscores the need for continued research to fully explore this therapeutic approach. The proposed treatment strategy, which combines the autoimmune hypothesis, the proliferative effects of GLP-1, and albumin ligand modifications, aims to restore beta cell mass and function, thereby improving the quality of life for individuals with type 1 diabetes. Clinical trials are planned for 2024 under the registration ‹Amr Ahmed, Maher M. Akl, Semaglutide GLP1 Agonists with Degludec Basal-bolus Insulin in Early Type 1 Diabetes to Basalbolus› (ClinicalTrials.gov Identifier NCT06057077).

## Introduction

 Type 1 diabetes is characterized by the destruction of beta cells, which necessitates the use of daily basal-bolus insulin as the primary treatment option. However, the emergence of drugs such as glucagon-like peptide 1 (GLP-1) agonists has brought new hope for the management of obesity and type 2 diabetes mellitus.^1^ Notably, the United States Food and Drug Administration (FDA) has approved the use of GLP-1 agonists for adolescents as young as 12 years old, making it a promising choice for various indications, including combating obesity at an early age.^2^ While the prolonged and stable effects of degludec (Deg), basal insulin, are primarily attributed to its mechanism of action involving binding to albumin, it is crucial to consider the factors contributing to its stability and extended duration of effects.^3^

 In this manuscript, we aim to address a letter to the editor titled “Semaglutide in Early Type 1 Diabetes” *by Paresh Dandona, M.D., Ph.D., and Ajay Chaudhur, M.D., published in The New England Journal of Medicine on September 7, 2023*. The authors conducted a retrospective analysis to evaluate the efficacy of semaglutide, a GLP-1 agonist, in patients with early type 1 diabetes.

 Their study included 10 patients aged between 21 and 39 who initiated semaglutide treatment within 3 months of their type 1 diabetes diagnosis. The patients were followed up for 12 months, and metabolic outcomes were analyzed. Some patients presented with diabetic ketoacidosis at the time of diagnosis, while others exhibited symptoms such as polyuria, polydipsia, and weight loss. Additionally, most patients had antibodies against glutamic acid decarboxylase. Semaglutide was administered initially at a weekly dose of 0.125 mg, and the dose of prandial insulin was gradually reduced while the semaglutide dose was increased. Within 3 months, prandial insulin was completely eliminated in all patients, and within 6 months, basal insulin was eliminated in 7 patients. Throughout the 12-month follow-up period, the mean glycated hemoglobin level decreased to 5.7-5.9%, the fasting C-peptide level increased, and the time spent in the target glucose range improved. The authors compared their findings with control groups from four studies involving patients with early type 1 diabetes who were receiving insulin therapy. The results revealed that initiating semaglutide soon after the diagnosis of type 1 diabetes was associated with the elimination of prandial and basal insulin, increased C-peptide levels, and improved glycemic control. However, as this was a retrospective analysis and lacked a control group from their own study, the authors emphasized the need for prospective, randomized clinical trials with larger sample sizes to further investigate this approach. Despite its limitations, this small case series suggests the potential of semaglutide as a treatment option in early type 1 diabetes. The objective of this manuscript is to support and interpret this approach and propose an alternative approach that may be more effective.^4^

###  Interpretation and Commentary on the Manuscript “ Semaglutide in Early Type 1 Diabetes” by Paresh Dandona, M.D., Ph.D., and Ajay Chaudhur, M.D., published in The New England Journal of Medicine on September 7, 2023.

**Comment 1**: The presence of anti-GAD antibodies in most cases of type 1 diabetes suggests an autoimmune etiology. Early intervention with GLP-1 agonists may potentially protect residual beta cells and reverse the autoimmune response. It is suggested that GLP-1 agonists like semaglutide may discourage the release of these antibodies, leading to the reversal of beta cell dysfunction. The reversible binding of degludec to albumin allows for the release of insulin in the target tissue, resulting in glucose-lowering effects. Further investigations, such as measuring anti-GAD and anti-IA2 antibodies after stopping insulin, can provide valuable insights into the validity of this theory.^5^

**Comment 2**: GLP-1 receptors are found in pancreatic islets, acini, and ducts. GLP-1 agonist therapy has been observed to stimulate proliferation in acinar and duct cells containing endocrine stem cells. Additionally, previous studies have suggested that pancreatic exocrine duct cells have the potential to differentiate into insulin-producing beta cells during embryogenesis but not after birth. The direct reprogramming of liver cells into insulin-producing cells offers another approach for cell replacement therapy.

 The plasticity of adult human hepatic tissue and its ability to acquire pancreatic characteristics have been demonstrated in vitro. These findings highlight the potential of utilizing adult hepatic tissue for regenerative medicine approaches.^6^

**Comment 3**: Modifying GLP-1 receptor agonists with albumin ligands specific for human serum albumin (HSA) has shown promise in extending the half-life of GLP-1 and enhancing its anti-diabetic effects. Rhein-C12-GLP-1, a derivative modified with Rhein, has demonstrated improved glucose tolerance and significant hypoglycemic effects in animal models. This modification could potentially increase the action time of GLP-1 and make it a viable option for the treatment of type 2 diabetes mellitus and as a long-acting anorectic agent. Further studies are needed to fully evaluate the potential of this modification, including molecular docking studies, pharmacokinetic assays, and pharmacology assays.^7^

###  Perspective

 The information provided suggests a comprehensive and interconnected perspective on the potential treatment of type 1 diabetes through the synergistic approach of dual GLP-1 agonist with degludec basal insulin. Type 1 diabetes is characterized by the destruction of beta cells, resulting in the need for daily basal-bolus insulin as the primary treatment option. However, the emergence of GLP-1 agonists offers new hope for the treatment of obesity and type 2 diabetes. These agonists have been approved by the FDA for adolescents as young as 12 years old, making them a promising choice for various indications, including combating obesity at an early age. The stability and prolonged effects of degludec basal insulin are attributed to its mechanism of action, which involves reversible binding to albumin. This allows for the release of more than 99% of the drug in the target tissue, exerting glucose-lowering effects. Notably, changes in albumin concentrations are unlikely to impact the pharmacokinetic properties of degludec due to the high levels of serum albumin. The bound insulin decreases at night, leading to increased free insulin levels and maintaining a constant association constant (Ka). This may result in a stronger effect of degludec at night, potentially causing nocturnal hypoglycemia.^8^

 Another intriguing approach discussed is the direct reprogramming of liver cells into insulin-producing cells, which could be a viable option for cell replacement therapy in diabetes. Experimental studies have shown that human liver cells possess significant cellular plasticity and can acquire mesenchymal-like characteristics.^9^ Insulin-producing cells were primarily generated in cells enriched for adult hepatic markers that coexpress albumin and mesenchymal markers.

 These findings suggest that adult human hepatic tissue retains a considerable level of developmental plasticity, which could be harnessed for regenerative medicine approaches.^10^ Furthermore, modifying GLP-1 receptor agonists with albumin ligands specific for HSA has proven to be an effective strategy for extending the half-life of GLP-1. This modification enhances the anti-diabetic profile of GLP-1, resulting in improved glucose tolerance and significant hypoglycemic effects.^11^ Rhein-C12-GLP-1, a derivative modified with Rhein, has shown promising hypoglycemic effects in animal models, surpassing the action time of the backbone peptide Arg34-GLP-1(7−37)-OH.^12^ It holds potential for the treatment of type 2 diabetes mellitus and could be developed as a long-acting anorectic agent. However, further evaluations, including molecular docking studies, pharmacokinetic assays, and pharmacology assays, are necessary to fully assess its potential. Evidence supports the autoimmune hypothesis as the basis for type 1 diabetes, characterized by the destruction of pancreatic beta cells and subsequent insulin deficiency.^13^ This hypothesis suggests that self-reactive T cells mistakenly target and attack beta cells, leading to their destruction. Autoantibodies against beta cell antigens, such as insulin, glutamic acid decarboxylase (GAD), and islet antigen-2 (IA-2), found in individuals with type 1 diabetes, serve as markers for the autoimmune process and provide insights into the disease’s pathogenesis.^14^

 GLP-1, a peptide hormone secreted by intestinal L-cells, plays a critical role in glucose homeostasis.^15^ Apart from its insulinotropic effects, GLP-1 has demonstrated the ability to promote cell proliferation and neogenesis in various tissues, including the pancreas.^16,17^ Studies have shown that activation of GLP-1 receptors stimulates the replication and survival of beta cells.^18^ This proliferative effect of GLP-1 on beta cells holds significant potential for the development of innovative therapeutic approaches in treating early type 1 diabetes, with the aim of restoring beta cell mass and function.^19^ To optimize the pharmacokinetic properties of GLP-1 and extend its duration of action, researchers have explored modifications using albumin ligands.^20^ Albumin, the most abundant protein in the blood plasma, has a long half-life and can reversibly bind to various drugs and hormones. By conjugating GLP-1 with specific albumin ligands, such as fatty acids or other chemical moieties, modified GLP-1 analogs exhibit enhanced binding affinity to albumin and prolonged circulation time.^21^ The use of albumin ligands in modification offers several advantages. Firstly, it improves the stability and pharmacokinetic profile of the GLP-1 analog by reducing renal clearance and enzymatic degradation.^22-24^ Secondly, the reversible binding of the modified GLP-1 analog to albumin facilitates its release at the target tissue, ensuring sustained therapeutic effects.^25^ Additionally, the high levels of serum albumin act as a buffer against changes in albumin concentrations, minimizing potential variations in pharmacokinetics.^26^

## Conclusion

 The proposed theory of beta cell regeneration in early type 1 diabetes through the synergistic approach of dual GLP-1 agonist with degludec basal insulin presents a comprehensive and interconnected perspective. This approach takes into account the unique pharmacokinetic properties of degludec, the potential plasticity of adult human hepatic tissue, and the promising outcomes of GLP-1 derivatives modified with albumin ligands. By combining these elements, there is potential for a transformative treatment strategy for type 1 diabetes. The autoimmune hypothesis, which supports the involvement of self-reactive T cells in the pathogenesis of type 1 diabetes, provides substantial evidence for the underlying mechanisms of the disease. Understanding the role of these cells in the destruction of beta cells is crucial for developing effective interventions. Moreover, the proliferative effects of GLP-1 on beta cell replication offer promise for novel therapeutic interventions in type 1 diabetes. By stimulating beta cell replication and enhancing their survival, GLP-1 holds the potential to restore beta cell mass and function, which is essential for glucose homeostasis. Additionally, modifications using albumin ligands offer a strategy to optimize the pharmacokinetic properties of GLP-1.

 By conjugating GLP-1 with albumin-specific ligands, the resulting modified GLP-1 analogs exhibit enhanced binding affinity to albumin, prolonging their circulation time and improving stability. This approach has the potential to provide sustained therapeutic effects and minimize potential pharmacokinetic variations. Despite these advancements, further research and clinical investigations are necessary to fully explore the potential of these approaches in managing type 1 diabetes. Continued efforts in understanding the autoimmune basis of the disease, refining GLP-1 analogs, and evaluating their efficacy in clinical settings will be crucial in advancing the field. The present hypothesis has been registered for undergoing clinical trials in 2024 under the title ‘Amr Ahmed, Maher M. Akl, Semaglutide GLP1 Agonists With Degludec Basal-bolus Insulin in Early Type 1 Diabetes to Basal-bolus.’ Additionally, the ClinicalTrials.gov Identifier for this study is NCT06057077.

## Competing Interests

 The authors declare that there are no conflicts of interest.

## Ethical Approval

 Not applicable.
